# Investigating casual association among gut microbiome and esophageal cancer: A Mendelian randomization study

**DOI:** 10.1097/MD.0000000000041563

**Published:** 2025-02-21

**Authors:** Falide Atabieke, Ailikamu Aierken, Munire Aierken, Mayinuer Rehaman, Qi-Qi Zhang, Jian Li, Yu Xia, Yierzhati Aizezi, Diliyaer Dilixiati, Hong-Liang Gao, Zhi-Qiang Zhang

**Affiliations:** a The Second Department of Gastroenterology, The First Affiliated Hospital of Xinjiang Medical University, Urumqi, China; b Graduate School of Xinjiang Medical University, Urumqi, China; c Department of Disinfection and Vector Biocontrol, Disease Prevention and Control Center, Urumqi, China; d Department of Physiology, School of Basic Medical Sciences, Xinjiang Medical University, Urumqi, China; e Center of Critical Care Medicine, First Affiliated Hospital of Xinjiang Medical University, Urumqi; f Department of Urology, The First Affiliated Hospital of Xinjiang Medical University, Urumqi, China.

**Keywords:** Asians, causality, esophageal cancer, gut microbiota, Mendelian randomization

## Abstract

The gut microbiota has been strongly linked to gastrointestinal cancer, but the relationship between gut microbiota and esophageal cancer (EC) is still not fully understood. We conducted a 2-sample Mendelian randomization (MR) study to unveil the potential impact of intestinal microorganisms on EC in East Asian populations. In order to delve deeper into the potential causal relationship between gut microbiota and EC, we conducted a 2-sample MR analysis, utilizing 211 single nucleotide polymorphisms associated with gut microbiota, sourced from the largest genome-wide association study on gut microbiota, for our analysis. To estimate the causal relationship, we employed the inverse variance weighting method. In addition, to assess the potential influence of pleiotropy, we used MR-Egger regression in our analysis. Among the 10 specific bacterial taxa identified using the inverse variance weighting as being associated with the risk of EC, we observed a positive association between family Bacteroidaceae (*P* = .04), genus *Bacteroides* (*P* = .04), genus *Bilophila* (*P* = .02), genus *Candidatus Soleaferrea* (*P* = .02) and the EC, while family Victivallaceae (*P* = .03), genus *Eubacterium coprostanoligenes* (*P* = .01), genus *Catenibacterium* (*P* = .01), genus *Coprococcus2* (*P* = .01), unknowngenus.id.959 (*P* = .02) and unknowngenus.id.1868 (*P* = .01) may be associated with a reduced risk of EC. Our MR analysis indicate a probable association between gut microbiota and the development and advancement of EC. These findings offer novel perspectives on the possible application of targeted gut bacteria for the prevention and management of EC.

## 1. Introduction

Esophageal cancer (EC) is a significant global health concern, ranking as the eighth most prevalent cancer worldwide. It is also the sixth leading cause of cancer-related deaths.^[1]^ The high mortality rate associated with EC makes it an important health concern even though it is not the most common cancer type. The 2 main histological subtypes of EC are adenocarcinoma and squamous cell carcinoma^[2]^ and a comprehensive evaluation and management approach is typically necessary for the majority of patients.^[3]^ Although many treatments like surgery, chemotherapy, radiation, and chemo-radiation have been developed, the outlook for patients remains bleak, even for those who have had all of the cancer removed with surgery.^[4]^ Therefore, it is essential to reveal the cause of EC.

The human gastrointestinal (GI) tract contains a diverse and dynamic population of microorganisms, collectively referred to as the gut microbiome. This intricate ecosystem plays a crucial role in influencing human health and disease.^[5]^ In 2008, the Human Microbiome Project, funded by the National Institutes of Health Common Fund in the United States, successfully isolated and sequenced over 1300 reference strains from the human body.^[6]^ According to the previous studies, the normal gut microbiota influence not only GI health but also systemic immunity, metabolism, and even neurological function.^[7–9]^ On the other hand, empirical evidence suggests that the human gut microbiome has the capacity to exert influence on the onset and progression of GI cancers as well.^[10]^ In terms of the esophagus, the gut microbiota may potentially exert significant influence on esophageal physiology, such as inducing mesenchymal transformation of esophageal epithelium and enhancing glucose uptake metabolism.^[11]^ Also, imbalances or disruptions in the intricate interplay between the gut microbiome and the esophagus, often caused by changes in their relative abundance, can contribute to the development of esophageal diseases, including EC.^[12,13]^ Yet, the presence of confounding factors makes it challenging to establish a definitive causal relationship with certainty.

Mendelian randomization (MR) is an epidemiological method utilized to evaluate the causal association between modifiable risk factors and their resulting outcomes.^[14]^ This technique leverages the random assortment of genes from parents to offspring that occurs during the formation of gametes and conception. Because the variants in our DNA (genetic polymorphisms) that we inherit are determined randomly, they can serve as proxies or instrumental variables for environmental exposures or modifiable risk factors. This allows researchers to overcome some of the limitations of observational studies, such as confounding factors and reverse causation. Several MR studies have effectively elucidated significant causal relationships between the gut microbiota and GI diseases. For instance, Long^[15]^ discovered a causal association between the genus *Tyzzerella3* and the genus *Ruminococcus torques* with colorectal cancer. Nevertheless, the causal relationship between the gut microbiome and EC remains unclear. By conducting a MR study, we can gain deeper insights into the matter, potentially contributing to more effective treatments for patients with EC.^[16]^

## 2. Materials and methods

### 2.1. Data sources

The MiBioGen International Consortium supplied the genetic research data on the gut microbiome. This study, which is currently the most comprehensive on microbiome-genome associations, analyzed the whole-genome genotypes and 16S ribosomal RNA (rRNA) fecal microbiome data from 18,340 individuals across 24 different populations. These populations are spread across the United States, Canada, Israel, South Korea, Germany, Denmark, the Netherlands, Belgium, Sweden, Finland, and the United Kingdom. The primary objective of the study is to understand the influence of autosomal genetic variations on the gut microbiome’s composition. The study encapsulated 211 taxa, which included 131 genera, 35 families, 20 orders, and 16 classes. EC outcome Genome-Wide Association Studies (GWAS) summary statistics were retrieved from the BioBank Japan (BBJ), a platform that facilitates open GWAS and encompasses 1388 cases along with 159,201 controls.

### 2.2. Study design

**Figure 1.**
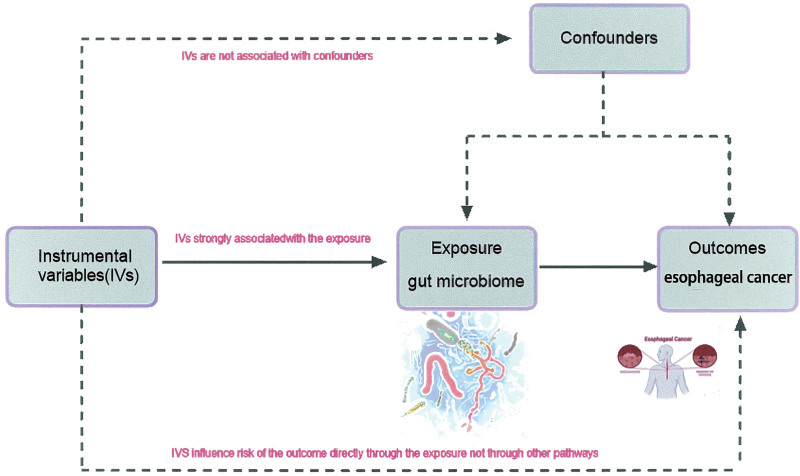
Conceptual framework for the Mendelian randomization analysis.

We executed a 2-sample MR study, leveraging GWAS data pertinent to the gut microbiome (https://mibiogen.gcc.rug.nl) and EC (https://pheweb.jp/downloads). Before initiating the study, we procured informed consent from the original publications and public databases, also ensuring the study received ethical approval. In our MR analysis, we relied on 3 crucial assumptions (Fig. 1): the instrumental variables used in the analysis must demonstrate a strong association with the gut microbiome; these variables should not be linked with any confounding factors; and the instrumental variables should induce effects exclusively by influencing the gut microbiome.^[17]^ Details are shown in Figure 1.

### 2.3. Selection of tool variables

Our initial step involved identifying single nucleotide polymorphisms (SNPs) that exhibited a strong association with exposure, signified by a *P* value less than 5 × 10^−8^. Given the scarcity of SNPs of genome-wide significance, we adopted a more relaxed threshold (*P *< 1 × 10^−5^) for SNP identification. We then utilized the Asian population reference data from the Human Genome Project^[18]^ to discern independent SNPs, adhering to a linkage disequilibrium *r*^2^ threshold of *r*^2^ < 0.001 and kb > 10,000. Subsequently, we examined the robustness of each instrumental variable by calculating the *F*-statistic, using the expression *F* = *R*^2^ × (*N*−2)/(1−*R*^2^).^[19]^ Here, *N* stands for the sample size of gut microbiota, while *R*^2^ denotes the percentage of gut microbiota variation explained by the selected SNP.^[20,21]^ SNPs with *F*-statistics exceeding 10 were chosen to mitigate instrumental variable bias.

Furthermore, we employed PhenoScanner V2(http://www.phenoscanner.medschl.cam.ac.uk/) to investigate the presence of potential confounding factors associated with these SNPs. Ultimately, to uphold the exclusivity assumption, we discarded SNPs (*P *< 5 × 10^−6^) that exhibited a direct and significant association with the outcome.

### 2.4. Statistical analyses

For our analysis, we employed 5 methodologies of MR: Inverse Variance Weighted (IVW), MR Egger, Weighted Median, Simple Mode, and Weighted Mode. Subsequently, the odds ratio (OR) values and 95% CI associated with these methodologies were visualized.

### 2.5. Sensitivity analysis

Our research employed a series of rigorous statistical tests to ensure the robustness and validity of our findings. Cochran Q test was performed to evaluate the presence of heterogeneity, with a significance level of .05 indicating its existence. The MR-Egger intercept test was utilized to examine the potential for horizontal pleiotropy, identified through a deviation of the estimated intercept from zero. To assess the sensitivity of our results, we conducted a leave-one-out analysis, which allowed us to speculate on the existence of outliers and assess the stability of our results. This was further supported by a review of the funnel plot outcomes. The impact of potential outliers on the results was identified and assessed using the Mendelian Randomization Pleiotropy RESidual Sum and Outlier (MR-PRESSO) method. The MR analysis techniques were carried out using the TwoSampleMR package (version 0.5.6) in R software (version 4.3.0).

### 2.6. Ethical audit

Relevant data were obtained from GWAS summary data (https://gwas.mrcieu.ac.uk/), BBJ (https://pheweb.jp/downloads) for publicly available data, informed consent and ethical approval was obtained from the original publications and these publicly available databases prior to conducting the study, which utilized the GWAS summary statistics to include SNPs associated with EC and gut microbiome in Asian pedigrees to be explored without the need for intramural ethical approval.

## 3. Results

### 3.1. The selection of tool variables

**Figure 2.**
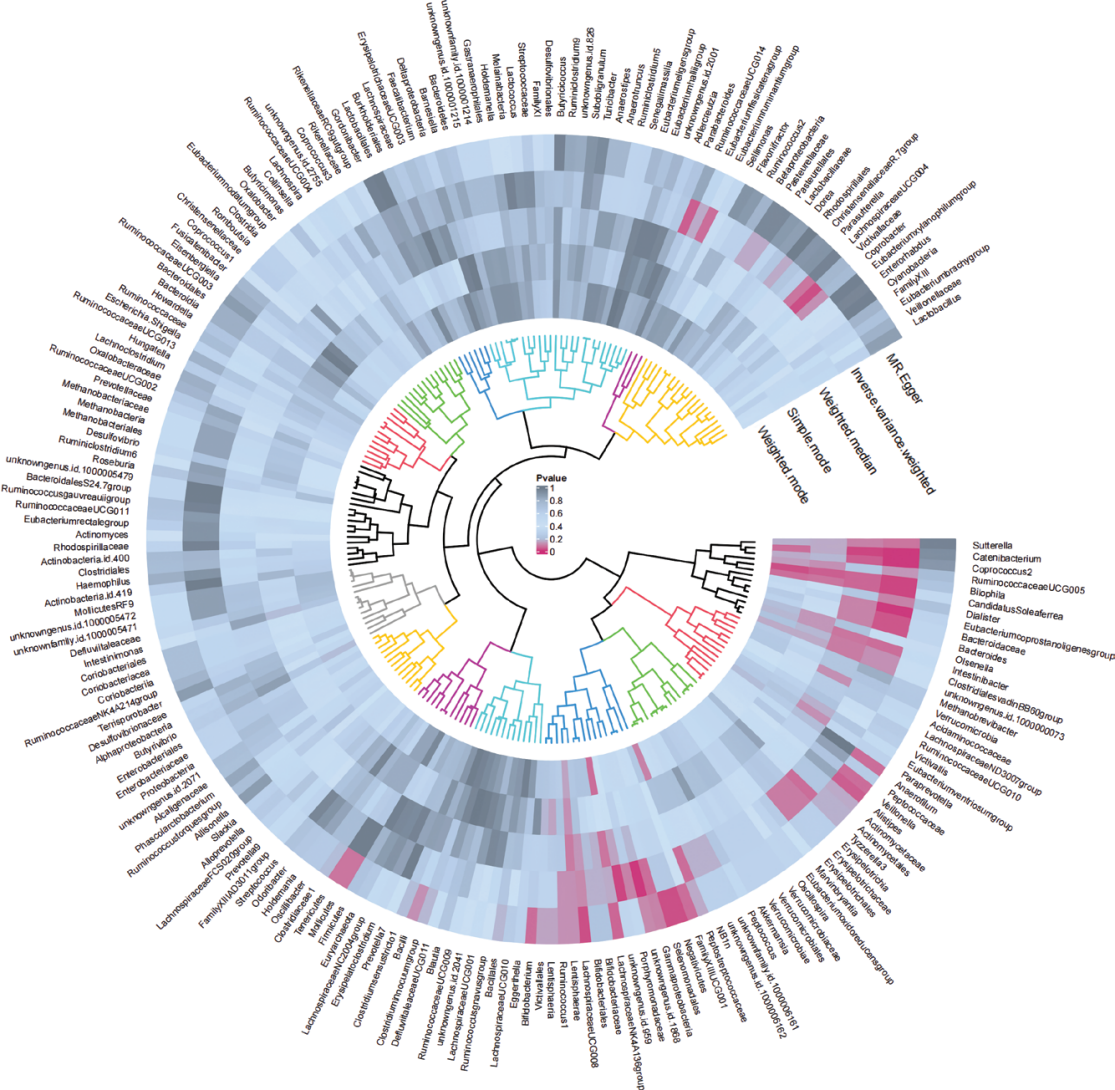
Circle diagram of 211 gut microbiomes on EC. EC = esophageal cancer.

In accordance with the predetermined selection criteria, an initial imbalance linkage test is undertaken to identify SNPs associated with both the gut microbiome and EC. Following this, SNPs demonstrating an *F*-statistic of <10 are excluded. The PhenoScanner database provides confirmation that no confounding factors are present. Subsequent outlier testing using the MR-PRESSO model revealed no anomalies. Ultimately, this rigorous process yielded 92 SNPs pertinent to the gut microbiome (Table S1, Supplemental Digital Content, http://links.lww.com/MD/O389). Furthermore, comprehensive statistical results for 211 gut microbiomes are available (Table S2, Supplemental Digital Content, http://links.lww.com/MD/O389, Fig. 2).

### 3.2. Two-sample MR analysis

**Figure 3.**
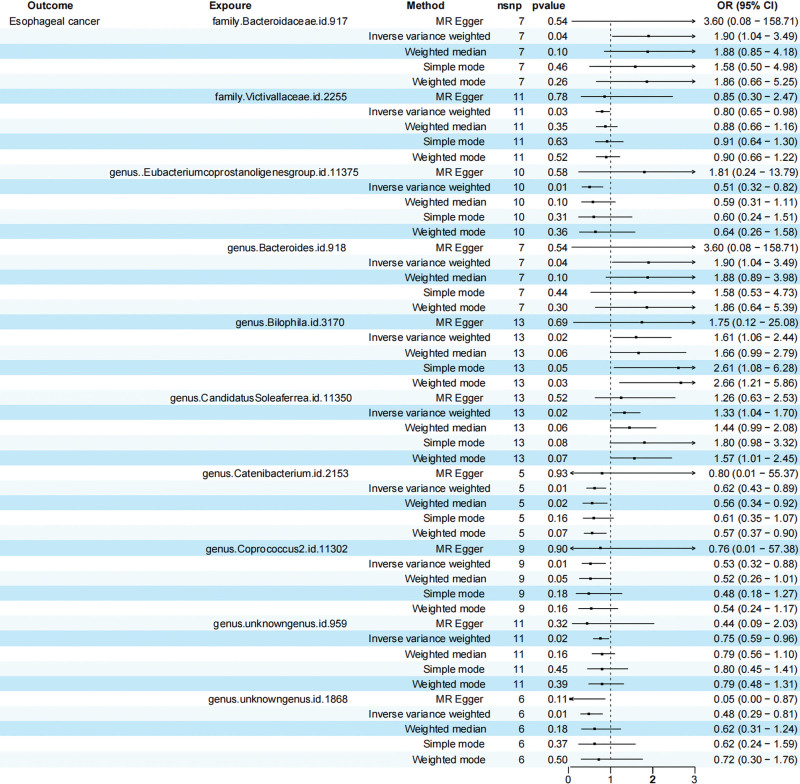
Forest plot of the relationship between 10 types of gut microbiota and the risk of EC. EC = esophageal cancer.

Our primary MR analysis, utilizing the IVW method, evaluated the correlation between the human gut microbiome and EC. The analysis revealed that the family Bacteroidaceae.id.917 (OR, 1.90 [95% CI, [1.04–3.49]; *P* = .04), genus.Bacteroides.id.918 (OR, 1.90 [95% CI, 1.04–3.49]; *P* = .04), and the genus.Bilophila.id.3170 (OR, 1.61 [95% CI, [1.06–2.44]; *P* = .02), genus.CandidatusSoleaferrea.id.11350 (OR, 1.33 [95% CI, [1.04–1.70]; *P* = .02) exhibit a positive correlation with EC (Table 1).Conversely, the family.Victivallaceae.id.2255 (OR, 0.80 [95% CI, [0.65–0.98]; *P* = .03), genus.Eubacteriumcoprostanoligenesgroup.id.11375 (OR, 0.51 [95% CI, [0.32–0.82]; *P* = .01), genus.Catenibacterium.id.2153 (OR, 0.62 [95% CI, [0.43–0.89]; *P* = .01), genus.Coprococcus2.id.11302 (OR, 0.53 [95% CI, 0.32–0.88]; *P* = .01), unknowngenus.id.959 (OR, 0.75 [95% CI, 0.59–0.96]; *P* = .02), and unknowngenus.id.1868 (OR, 0.48 [95% CI, 0.29–0.81]; *P* = .01) were found to be negatively correlated with EC. Table 1 and Figure 3 present a visual depiction of the obtained results.

**Table 1:** MR results of causal links between gut microbiome and EC risk (*P* < 1 × 10^−5^).

Classification	No. of SNP	Methods	β	SE	OR (95% CI)	*P* value	Horizontal pleiotropy Egger intercept	SE	*P* value	Heterog eneity Cochran Q	*P* value
family.Bacteroidaceae.id.917	7	MR Egger	1.28	1.93	3.60 (0.08–158.71)	.54	−0.03	0.12	0.75		
		Inverse variance weighted	0.64	0.31	1.90 (1.04–3.49)	.04				2.47	.87
		Weighted median	0.63	0.38	1.88 (0.89–4.00)	.10					
		Simple mode	0.46	0.58	1.58 (0.51–4.96)	.46					
		Weighted mode	0.62	0.50	1.86 (0.70–4.96)	.26					
family.Victivallaceae.id.2255	11	MR Egger	−0.16	0.54	0.85 (0.30–2.47)	.78	−0.01	0.07	0.9		
		Inverse variance weighted	−0.22	0.11	0.80 (0.65–0.98)	.03				7.22	.7
		Weighted median	−0.13	0.14	0.88 (0.66–1.16)	.35					
		Simple mode	−0.09	0.18	0.91 (0.64–1.30)	.63					
		Weighted mode	−0.10	0.16	0.90 (0.66–1.22)	.52					
genus.Eubacteriumcoprostanoligenesgroup.id.11375	10	MR Egger	0.59	1.04	1.81 (0.24–13.79)	.58	−0.08	0.07	0.24		
		Inverse variance weighted	−0.67	0.24	0.51 (0.32–0.82)	.01				8.65	.47
		Weighted median	−0.53	0.33	0.59 (0.31–1.11)	.10					
		Simple mode	−0.51	0.47	0.60 (0.24–1.51)	.31					
		Weighted mode	−0.45	0.46	0.64 (0.26–1.58)	.36					
genus.Bacteroides.id.918	7	MR Egger	1.28	1.93	3.60 (0.08–158.71)	.54	−0.04	0.12	0.75		
		Inverse variance weighted	0.64	0.31	1.90 (1.04–3.49)	.04				2.47	.87
		Weighted median	0.63	0.38	1.88 (0.89–3.98)	.10					
		Simple mode	0.46	0.56	1.58 (0.53–4.73)	.44					
		Weighted mode	0.62	0.54	1.86 (0.64–5.39)	.30					
genus.Bilophila.id.3170	13	MR Egger	0.56	1.36	1.75 (0.12–25.08)	.69	−0.01	0.09	0.95		
		Inverse variance weighted	0.48	0.21	1.61 (1.06–2.44)	.02				16.41	.17
		Weighted median	0.51	0.26	1.66 (0.99–2.79)	.06					
		Simple mode	0.96	0.45	2.61 (1.08–6.28)	.05					
		Weighted mode	0.98	0.40	2.66 (1.21–5.86)	.03					
genus.CandidatusSoleaferrea.id.11350	13	MR Egger	0.23	0.35	1.26 (0.63–2.53)	.52	0.01	0.04	0.89		
		Inverse variance weighted	0.28	0.12	1.33 (1.04–1.70)	.02				8.77	.72
		Weighted median	0.36	0.19	1.44 (0.99–2.08)	.06					
		Simple mode	0.59	0.31	1.80 (0.98–3.32)	.08					
		Weighted mode	0.45	0.23	1.57 (1.01–2.45)	.07					
genus.Catenibacterium.id.2153	5	MR Egger	−0.22	2.16	0.80 (0.01–55.37)	.93	−0.03	0.27	0.91		
		Inverse variance weighted	−0.48	0.19	0.62 (0.43–0.89)	.01				3.80	.43
		Weighted median	−0.58	0.25	0.56 (0.34–0.92)	.02					
		Simple mode	−0.49	0.28	0.61 (0.35–1.07)	.16					
		Weighted mode	−0.56	0.23	0.57 (0.34–0.90)	.07					
genus.Coprococcus2.id.11302	9	MR Egger	−0.28	2.21	0.76 (0.01–57.38)	.90	−0.03	0.16	0.88		
		Inverse variance weighted	−0.63	0.26	0.53 (0.32–0.88)	.01				6.79	.56
		Weighted median	−0.66	0.34	0.52 (0.26–1.01)	.05					
		Simple mode	−0.74	0.50	0.48 (0.18–1.27)	.18					
		Weighted mode	−0.62	0.40	0.54 (0.24–1.17)	.16					
genus.unknowngenus.id.959	11	MR Egger	−0.82	0.78	0.44 (0.09–2.03)	.32	0.06	0.09	0.50		
		Inverse variance weighted	−0.28	0.12	0.75 (0.59–0.96)	.02				10.56	.39
		Weighted median	−0.24	0.17	0.79 (0.56–1.10)	.16					
		Simple mode	−0.23	0.29	0.80 (0.45–1.41)	.45					
		Weighted mode	−0.23	0.26	0.79 (0.48–1.31)	.39					
genus.unknowngenus.id.1868	6	MR Egger	−2.91	1.41	0.05 (0.00–0.87)	.11	0.18	0.12	0.19		
		Inverse variance weighted	−0.73	0.26	0.48 (0.29–0.81)	.01				5.41	.37
		Weighted median	−0.47	0.35	0.62 (0.31–1.24)	.18					
		Simple mode	−0.47	0.48	0.62 (0.24–1.59)	.37					
		Weighted mode	−0.33	0.45	0.72 (0.30–1.76)	.50					

EC = esophageal cancer, MR = Mendelian randomization, OR = odds ratio, SE = standard error, SNP = single nucleotide polymorphism

The MR results, as computed via the IVW method, did not demonstrate noteworthy heterogeneity. Pleiotropy between the instrumental variables and the outcomes was assessed through MR-Egger regression, with the test results derived from the Egger-intercept method revealing no evidence of pleiotropy (Table 1). In the Outlier-test of the MR-PRESSO model, no outlier SNPs were detected, this suggests that the instrumental variables do not have a significant impact on outcome anomalies through pathways other than the exposure being studied. Consequently, no substantial evidence exists to affirm a pleiotropic relationship between these bacteria and EC. Comprehensive details concerning the instrumental variables mentioned above are available (Table S1, Supplemental Digital Content, http://links.lww.com/MD/O389). The *F*-statistic for all SNPs exceeds 10, implying the absence of weak instrument bias (Table S1, Supplemental Digital Content, http://links.lww.com/MD/O389).

The results derived from the leave-one-out sensitivity analysis underscore the model’s robust stability, with no anomalous SNPs identified. The scatter plots and results of the leave-one-out test are presented in the supplementary figures (Figures S1 to S10, Supplemental Digital Content, http://links.lww.com/MD/O390). To summarize, these findings provide strong evidence for a consistent and genetically driven causal relationship between gut microbiota and EC.

## 4. Discussion

In this study, in order to attain a more comprehensive understanding of the association among gut microbiota and EC, we analyzed the correlation and causality between the two. The findings from the MR suggest a correlation between gut microbiota and the occurrence and progression of EC.

A study conducted by Li et al^[22]^ concluded that distinct variations in fecal microbiota composition and higher abundance were observed between individuals with GI cancer and those in good health. Our findings align with Li et al and similar studies, suggesting an unknown relationship between gut microbiota and the occurrence of EC. Subsequently, through MR analysis of 211 microbial taxonomic units and EC, we identified 10 bacterial taxa that potentially exhibit causal associations with EC (all with *P *< .05). This work, utilizing MR analysis, provides a comprehensive exploration of the potential relationship between gut microbiota and EC for the first time.

The gut microbiota, once considered the “forgotten organ,” has emerged as a current research hotspot due to its significant associations with various pathological conditions, including thyroid diseases, gallbladder diseases, Alzheimer disease, polycystic ovary syndrome, and respective cancer.^[23–27]^ Fecal samples serve as a convenient and accessible source for studying. Recent research utilizing the analysis of 16S rRNA gene sequences extracted from fecal samples has provided evidence of distinct bacterial group enrichment and reduction patterns associated with specific diseases. Indeed, the role of the gut microbiota in GI diseases, including GI cancers, has been the subject of extensive research. Numerous studies have highlighted the strong correlation between the gut microbiota and these diseases.^[28]^ In a case-control study conducted by Sinha in 2016 found that the abundance of *Clostridium* and *Porphyromonas* was higher in fecal samples from colorectal cancer patients, while the abundance of *Fusobacterium* and *Spirochaetaceae* was lower.^[29]^ Furthermore, Liang study using 16S rRNA gene sequencing revealed that *Streptococcus* alterations were closely associated with gastric cancer, potentially serving as potential biomarkers for predicting gastric cancer.^[30]^ Although research on the gut microbiota and EC is relatively limited, some studies have identified the log-ratio of *Streptococcus* to *Veillonella* and *Streptococcus* to *Lactobacillus* as potential diagnostic biomarkers for esophageal squamous cell carcinoma, with receiver operating characteristic areas under the curve of 0.863 (95% CI, [0.707–1.000]) and 0.825 (95% CI, [0.673–0.977]), respectively.^[31]^ Yes, these findings strongly suggest that dysbiosis of the gut microbiota may be closely linked to an elevated risk of developing GI cancers.

The large intestine is the most densely populated and metabolically active site of the human body, primarily comprising anaerobic microbiota, Firmicutes, Bacteroidetes, Actinobacteria, Proteobacteria, and Verrucomicrobia.^[32]^ The family Bacteroidaceae is a bacterial family within the phylum Bacteroidetes, which includes the genus *Bacteroides* comprising numerous species. Members of the family Bacteroidaceae are typical gram-negative, obligate anaerobic bacteria that inhabit the GI tract of mammals.^[33]^ Studies have found significant dysbiosis in the gut microbiota of patients with EC, including a high prevalence of *Bacteroides vulgatus* and other *Bacteroides* species.^[23]^ In our research, we observed a positive correlation between the presence of heparinase and collagenase-producing *Bacteroides* and EC, which remained stable at the family and genus levels. Studies on Bacteroides and colorectal cancer suggest that the toxin produced by enterotoxigenic *Bacteroides fragilis* activates host-produced polyamine oxidase, generating hydrogen peroxide and reactive oxygen species, damaging DNA in epithelial cells, and promoting tumor development.^[34]^ Another study indicates that these bacteria may induce the production of the pro-inflammatory cytokine IL-17, thereby reducing the host’s anticancer immune response and allowing cancer growth.^[35]^ We also observed a positive correlation between genus *Bilophila* and genus *Candidatus Soleaferrea* and EC. Zhao MR study on the gut microbiota, involving 119 known genera, indicates that the presence of the *Bilophila* genus in the gut microbiota is associated with an increased risk of gastric and duodenal ulcers.^[36]^ Although there is limited research on these 2 bacteria specifically causing cancer, certain studies have indicated that specific strains of Bilophila may contribute to intestinal inflammation. They have the ability to produce harmful substances, including hydrogen sulfide, which can trigger inflammatory responses.

Our research also indicates a reduced risk of EC associated with family Victivallaceae, genus *Eubacterium coprostanoligenes*, genus *Catenibacterium*, and genus *Coprococcus2*. Among them, *Eubacterium* and *Ruminococcus* are well-recognized as major producers of short-chain fatty acids, particularly butyrate, in the human gut. These fatty acids possess antiinflammatory properties, helping to maintain a healthy gut environment. By promoting the integrity of the intestinal barrier and modulating immune responses, they help promoting gut health.^[37]^ In addition to butyrate production, Eubacteria, particularly *Eubacterium hallii*, *Eubacterium ramulus*, and *Eubacterium ventrosum*, also impact immune regulation, suppression of intestinal inflammation, and conversion of cholesterol and bile acids.^[38]^ Currently, little is known about the relationship between the gut microbiota and EC, and the association between family Lachnospiraceae, genus *Fusobacterium*, and EC has not been reported. Thus, our research offers a fresh perspective on comprehending the significance of the gut microbiota in EC, and further investigation is needed to explore the mechanisms by which these bacteria contribute to EC.

Based on current research, it has been found that the microbiota plays a significant role in promoting cancer development through a multitude of mechanisms, one of which involves influencing immune responses. In a study conducted by Münch et al,^[39]^ a mouse model of Barrett esophagus was utilized to explore the effects of a high-fat diet-induced alterations in gut microbiota. The findings revealed that these changes resulted in elevated immune cells. Ultimately, this led to the development of a pro-tumor immune phenotype, indicating a potential link between alterations in the gut microbiota and cancer progression. This study highlights the crucial role of the gut microbiota in mediating the effects of diet, specifically through inflammatory mechanisms. The study underscores the significance of maintaining a healthy microbiome in preventing or mitigating the inflammatory processes associated with diseases such as cancer. In a study conducted by Deshpande et al,^[40]^ it was discovered that bacteria capable of producing lactate were notably elevated in individuals with multiple GI diseases. In addition, certain pathogenic bacteria have the ability to produce genotoxins, which can stimulate cell proliferation and induce mutations.^[41]^ On the other hand, Li et al^[42]^ research has demonstrated that a diverse microbiota composition can prevent adverse immune reactions. Transplanting fecal matter from “healthy” mice into antibiotic-treated xenograft mice significantly improved liver metastasis, highlighting the protective role of the gut microbiota.^[43]^ Another study conducted by Wu et al^[44]^ revealed that specific members of the gut microbiota have a primarily protective function. Disruption and reduction of these beneficial bacteria have been associated with increased inflammatory responses and decreased survival time. While some evidence has been gathered, investigation into the relationship between the gut microbiota and esophageal adenocarcinoma is still relatively constrained in terms of available research. Whether the detection of fecal microbiota can explain the microbial characteristics of upper GI tumor patients is a question worth exploring. In recent years, an increasing number of studies have found that the gut microbiota also influences the incidence and progression of non-GI tumors.^[45,46]^ Therefore, we believe that the fecal microbiota may be of significant importance in further characterizing EC. Future research should focus on identifying potential mechanistic connections.

Despite our findings, there are still some limitations to our research. First, our data sources are primarily from East Asian populations. Therefore, it cannot be denied that our results may lead to estimated biases that affect generalizability. Second, in the current study, the analysis of bacterial taxa was limited to the family or genus level. However, it is worth noting that the utilization of GWASs in conjunction with advanced shotgun metagenomic sequencing analysis could potentially yield more specific and accurate results. By delving deeper into the genetic makeup of the microbiota, a finer resolution of bacterial taxa could be achieved, enhancing our understanding of their potential associations with the studied variables. Further exploration using advanced sequencing techniques may provide valuable insights into the complex interactions between microbial communities and their hosts. Third, the EC data from BBJ were not further subdivided into squamous cell carcinoma or adenocarcinoma, which means our study could only analyze the gut microbiota in relation to the overall EC population. Future research is hoped to provide more insights into the relationship between specific parts of EC and the gut microbiota. Meanwhile, recent studies suggest that future research should adopt a systemic approach and utilize various omics platforms to enhance our understanding of disease mechanisms in the context of complex gene-environment interactions.^[47,48]^

In general, we have conducted a comprehensive analysis of the relationship between EC and the gut microbiota. Our research results have identified a potential causal relationship between the gut microbiota and EC. This study may provide new insights into the mechanisms of EC development mediated by the gut microbiota.

## Author contributions

**Writing – original draft:** Falide Atabieke.

**Software:** Ailikamu Aierken, Jian Li, Yierzhati Aizezi.

**Formal analysis:** Munire Aierken, Yu Xia.

**Data curation:** Mayinuer Rehaman, Qi-Qi Zhang.

**Supervision:** Yierzhati Aizezi, Zhi-Qiang Zhang.

**Methodology:** Diliyaer Dilixiati, Hong-Liang Gao.

## Supplementary Material


