# Supplementary material for: Investigating casual association among gut microbiome and esophageal cancer: A Mendelian randomization study
Source: Medicine (Baltimore). 2025 Feb 21;104(8):e41563. doi: 10.1097/MD.0000000000041563 (PMC11856886; doi:10.1097/MD.0000000000041563)
Supplement: Supplementary file 2 [file medi-104-e41563-s002.pdf]

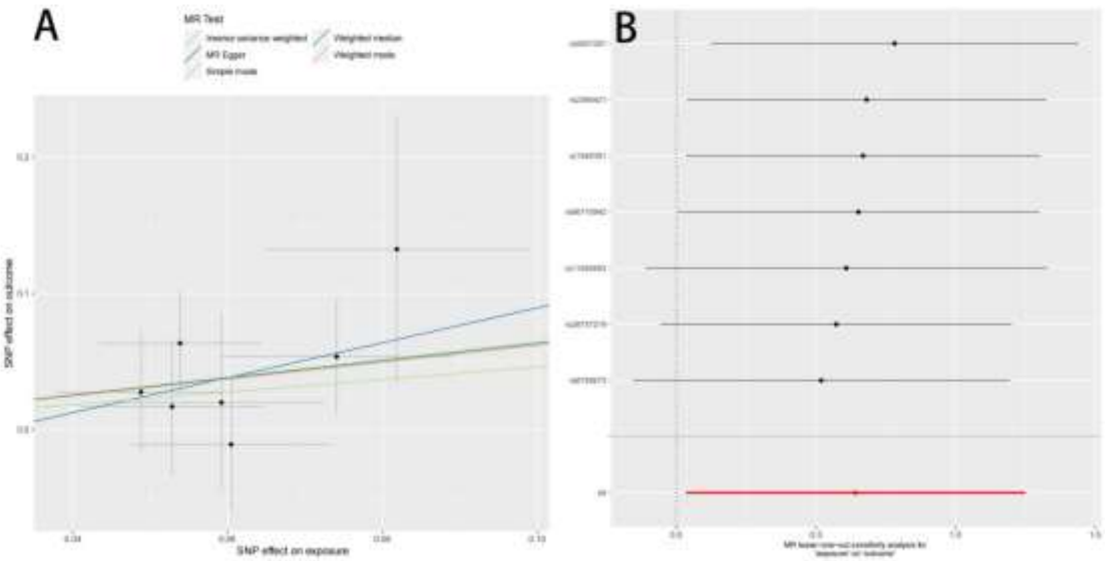

Figure S1.(A) Scatter plots for effect sizes of SNPs for *family.Bacteroidaceae* Esophageal cancer. (B) Leave-one-out sensitivity analysis for *family.Bacteroidaceae* Esophageal cancer.Abbreviations : SNP, single nucleotide polymorphism.

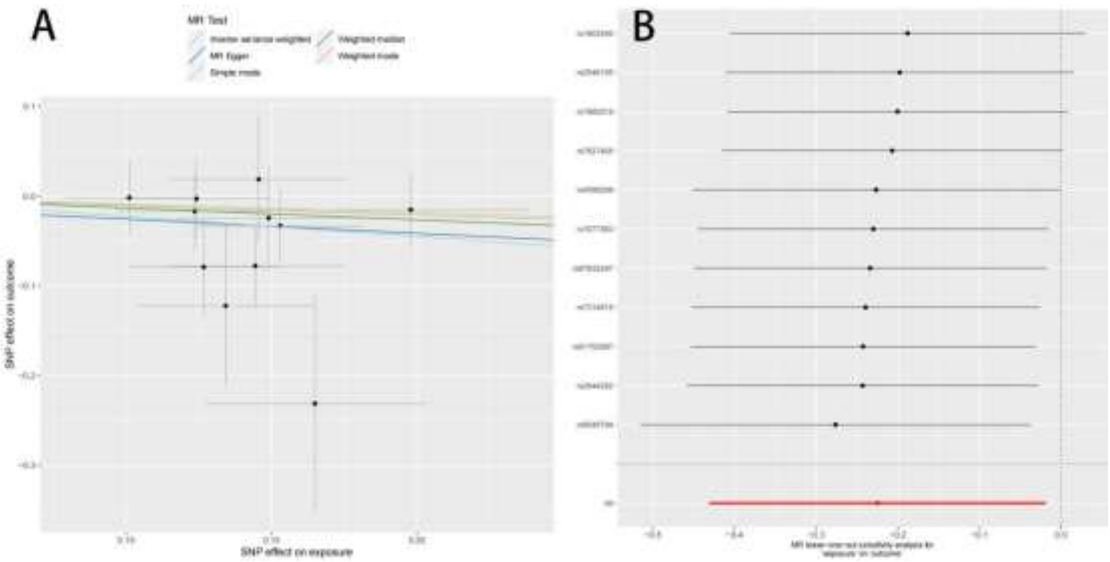

Figure S2.(A) Scatter plots for effect sizes of SNPs for *family.Victivallaceae.* Esophageal cancer. (B) Leave-one-out sensitivity analysis for *family.Victivallaceae.* Esophageal cancer.Abbreviations : SNP, single nucleotide polymorphism.

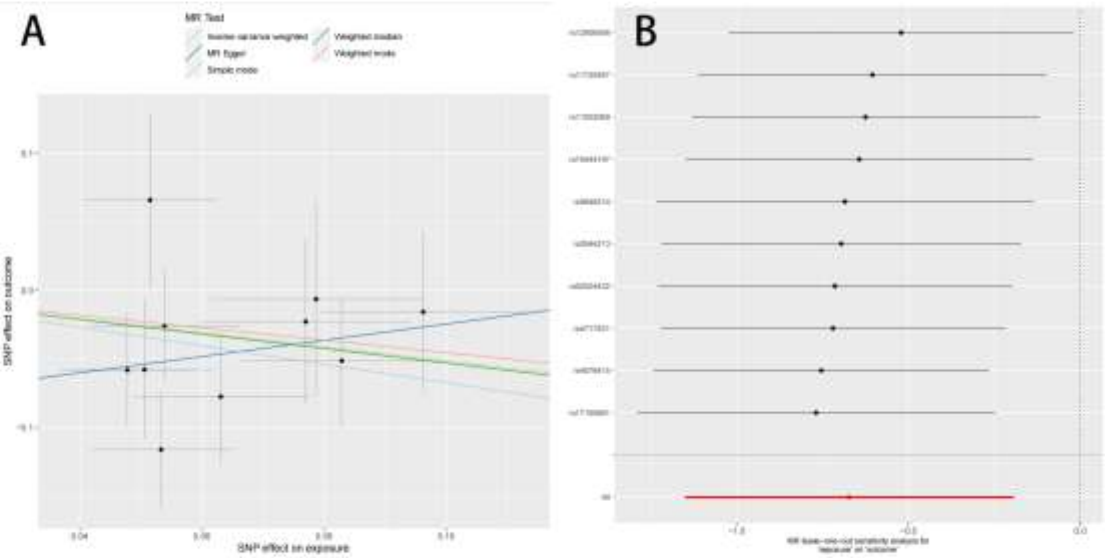

Figure S3.(A) Scatter plots for effect sizes of SNPs for *genus..Eubacteriumcoprostanoligenesgroup* Esophageal cancer. (B) Leave-one-out sensitivity analysis for *genus..Eubacteriumcoprostanoligenesgroup* Esophageal cancer.Abbreviations : SNP, single nucleotide polymorphism.

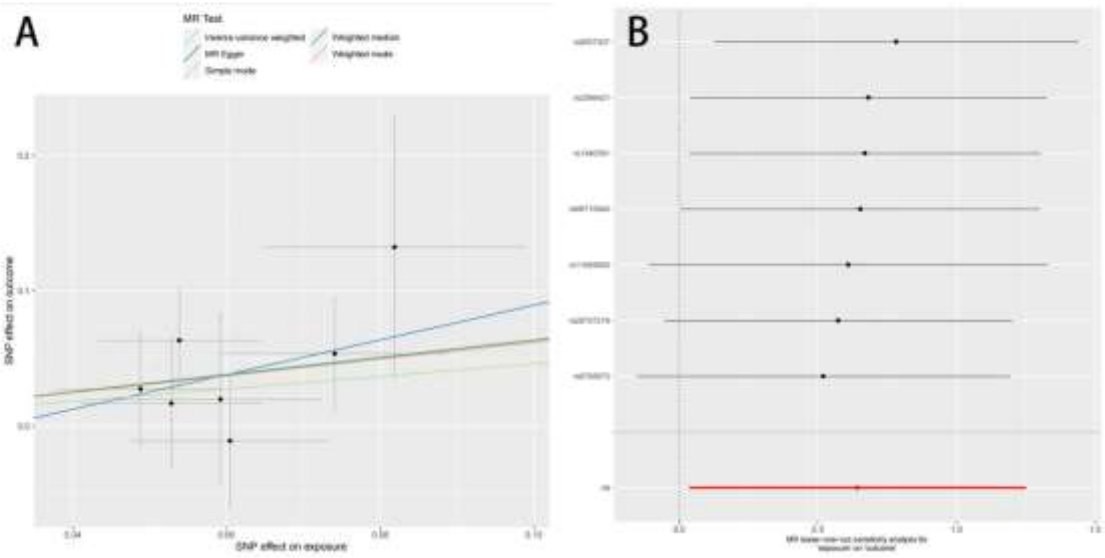

Figure S4.(A) Scatter plots for effect sizes of SNPs for *genus.Bacteroides* Esophageal cancer. (B) Leave-one-out sensitivity analysis for *genus.Bacteroides* Esophageal cancer.Abbreviations : SNP, single nucleotide polymorphism.

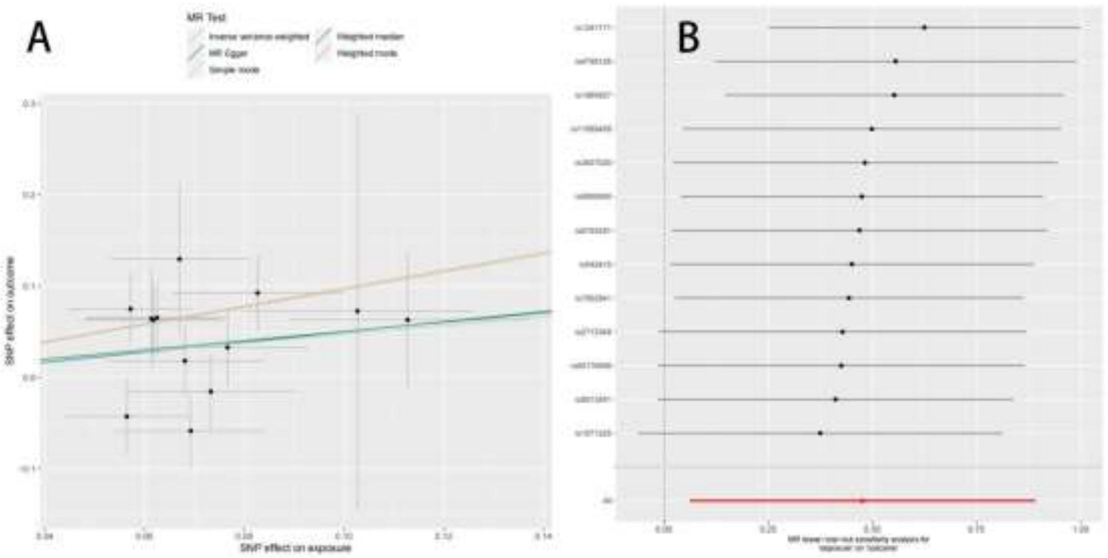

Figure S5.(A) Scatter plots for effect sizes of SNPs for *genus.Bilophila* Esophageal cancer. (B) Leave-one-out sensitivity analysis for *genus.Bilophila* Esophageal cancer.Abbreviations : SNP, single nucleotide polymorphism.

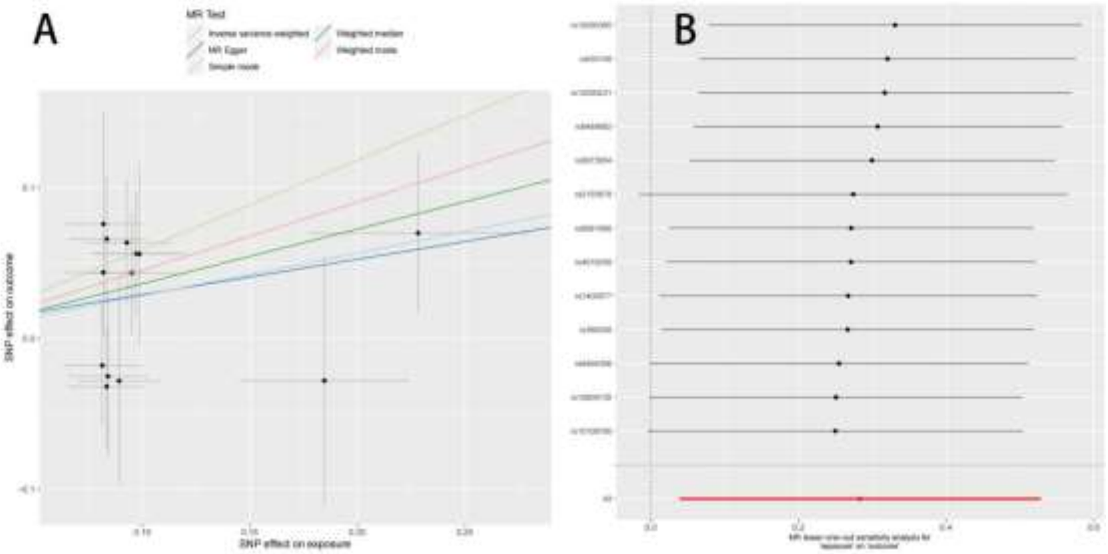

Figure S6.(A) Scatter plots for effect sizes of SNPs for *genus.CandidatusSoleaferrea* Esophageal cancer. (B) Leave-one-out sensitivity analysis for *genus.CandidatusSoleaferrea* Esophageal cancer.Abbreviations : SNP, single nucleotide polymorphism.

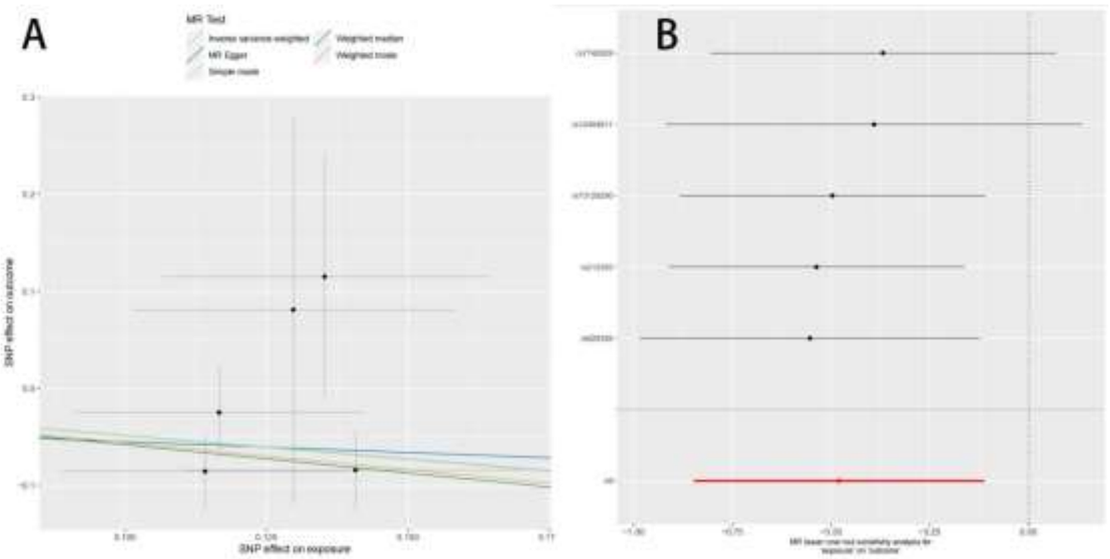

Figure S7.(A) Scatter plots for effect sizes of SNPs for *genus.Catenibacterium* Esophageal cancer. (B) Leave-one-out sensitivity analysis for *genus.Catenibacterium* Esophageal cancer.Abbreviations : SNP, single nucleotide polymorphism.

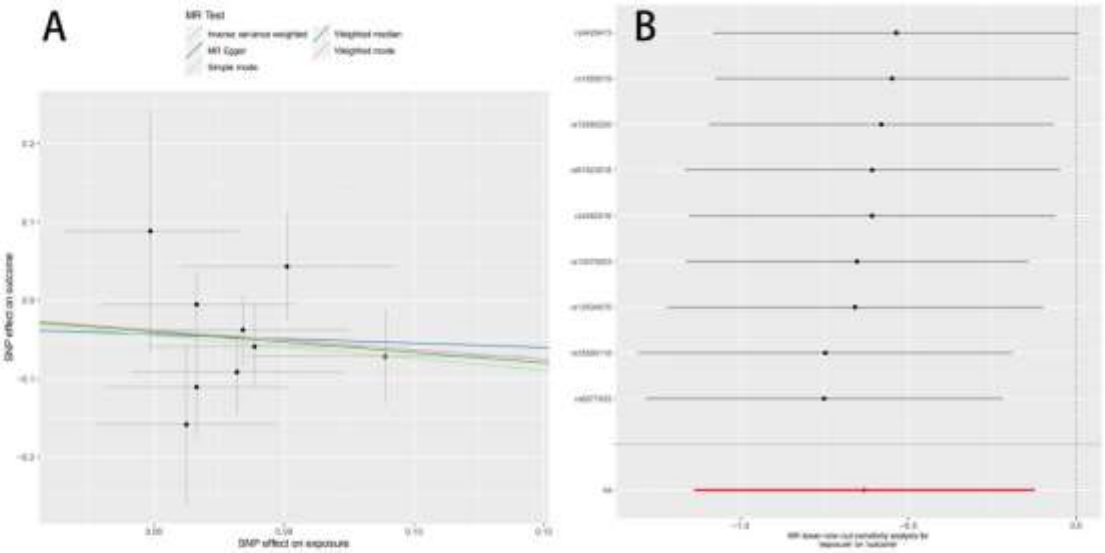

Figure S8.(A) Scatter plots for effect sizes of SNPs for *genus.Coprococcus2* Esophageal cancer. (B) Leave-one-out sensitivity analysis for *genus.Coprococcus2* Esophageal cancer.Abbreviations : SNP, single nucleotide polymorphism.

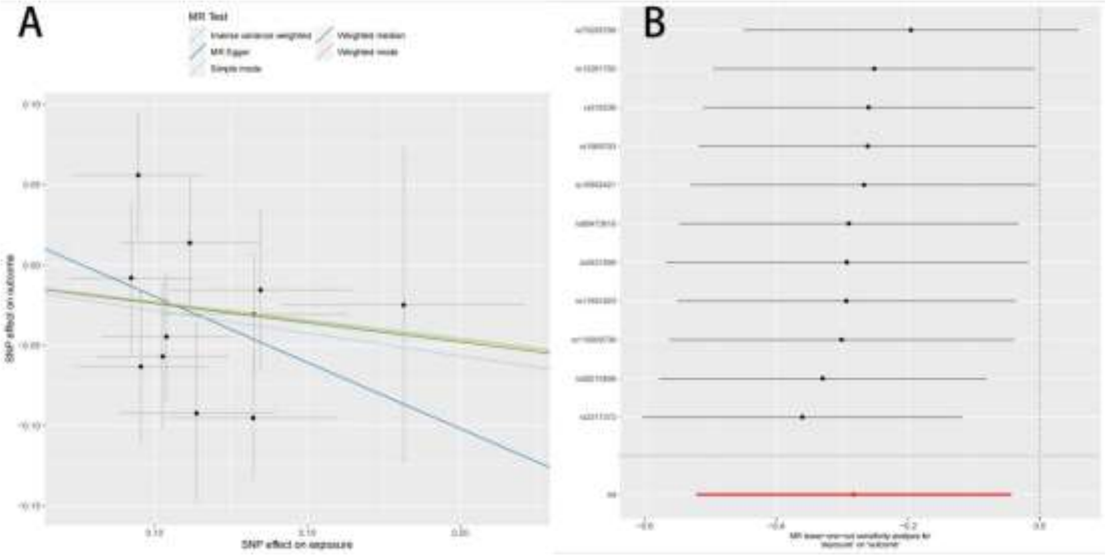

Figure S9.(A) Scatter plots for effect sizes of SNPs for *genus.unknowngenus.id.959* Esophageal cancer. (B) Leave-one-out sensitivity analysis for *genus.unknowngenus.id.959* Esophageal cancer.Abbreviations : SNP, single nucleotide polymorphism.

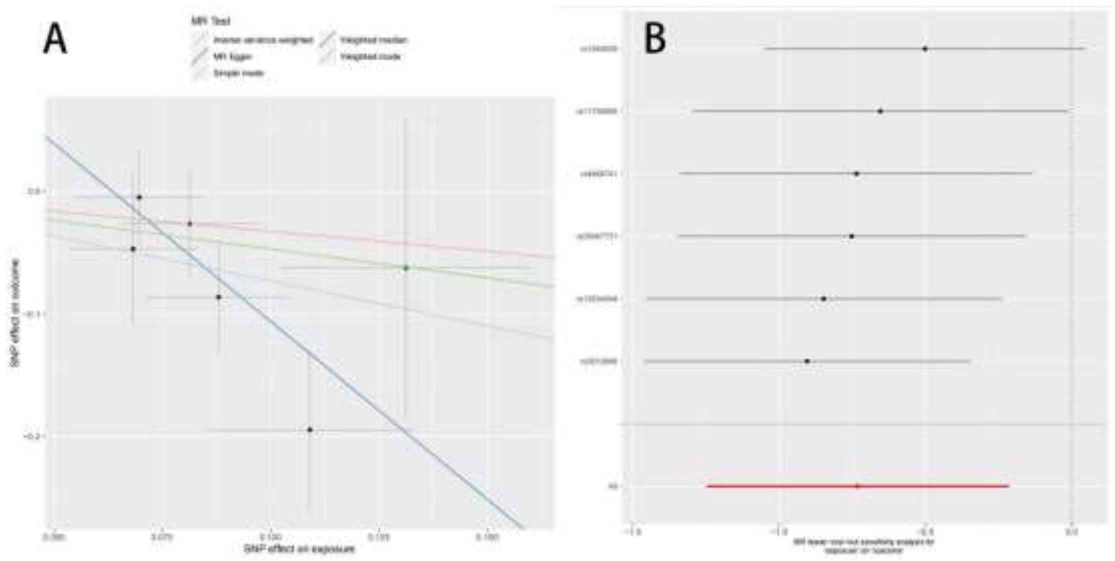

Figure S10.(A) Scatter plots for effect sizes of SNPs for *genus.unknowngenus.id.1868* Esophageal cancer. (B) Leave-one-out sensitivity analysis for *genus.unknowngenus.id.1868* Esophageal cancer.Abbreviations : SNP, single nucleotide polymorphism.
